# A power-free, parallel loading microfluidic reactor array for biochemical screening

**DOI:** 10.1038/s41598-018-31720-y

**Published:** 2018-09-12

**Authors:** Yanwu Liu, Gang Li

**Affiliations:** 1 College of Stomatology, Chongqing Medical University, Chongqing, 401147 China; 2 Defense Key Disciplines Lab of Novel Micro-Nano Devices and System Technology, Key Laboratory of Optoelectronic Technology and Systems, Ministry of Education, Chongqing University, Chongqing, 400044 China

## Abstract

This paper presents a power-free, self-contained microfluidic device in which a number of nanoliter-sized droplets can be parallelly and accurately metered and mixed for high-throughput analysis and/or portable systems. In this system, the absorption of air by pre-degassed PDMS and the change of capillary force due to sudden narrowing of the channel cross-section provide the mechanism for actuating, metering and mixing the flow of fluid in the microfluidic channels and chambers. With an array of channels and capillary valves combined with an array of pre-degassed PDMS pump chambers, the device can perform multiple liquid dispensing and mixing in parallel, and its performance and reproducibility are also evaluated. As a practical application, the proposed device is used to screen crystallization conditions of lysozyme. This device needs neither external power nor complex instruments for fluid handling. Thus, it offers an easy-to-use, inexpensive and power-free way to perform multiple nanoliter-volume distinct reactions in parallel format and should be ideally suitable for individual laboratories for various applications such as enzyme assay, protein crystallization, drug discovery, and combinatorial chemistry.

## Introduction

As the fields of combinatorial chemistry and high-throughput screening (HTS) have been growing, it has become increasingly desirable to develop the capabilities of rapidly and reliably carrying out chemical and biochemical reactions in large numbers using small quantities of samples and reagents^1^. Currently, the standard HTS method depends on microtitre technology, which typically involves microplates and pipettes to dispense and mix selected liquid samples and reagents to perform chemical or biochemical reactions in parallel format. However, this microplate-based system is burdened by several issues such as high reagent/sample consumption, laborious manual pipetting transfer and uncontrolled liquid evaporation. Although a few commercially available dispensing robots have been introduced to allow rapid and accurate dispensing of very small liquid volumes, these robotic systems have obvious limitations in terms of miniaturization and integration. Moreover, current robotics-based HTS methods are generally inaccessible to academic investigators due to significant instrument and maintenance costs. The emergence of microfluidic technology offers a powerful tool for the HTS applications. The advantages of microfluidics-based HTS systems include less sample/reagent consumption, lower operating costs, less reaction time, better portability, and higher reliability^2–6^. Despite these advantages, there are still some challenges facing these systems that need to be addressed. One major challenge in producing a microfluidics-based HTS system is the development of reliable microfluidic manipulation techniques. Currently, most microfluidic systems still require external macroscopic actuators, cumbersome fluidic connections, and electromechanical interfaces for liquid operation, which limit their miniaturisation and portability. Although there have been various types of microactuator-integrated microfluidic devices developed for portable applications utilizing piezoelectric^7,8^, electrostatic^9^, pneumatic^10,11^, or magnetic effects^12,13^, the integrated microfluidic devices with actuation components (*e.g*., diaphragms, actuators, valves, and heaters) have certain inherent disadvantages such as high costs, complex fabrication/assembly and complex control circuitry. To avoid costly and non-disposable integrated control components, a number of simple liquid-handling techniques without external power requirements have been developed, such as surface tension-based passive pumping^14,15^, evaporation-based passive pumping^16–18^, capillary flow^19–21^, gravity-driven flow^22,23^, and finger-actuated pumping^24–26^. These portable liquid-handling techniques address some important challenges in the design of microfluidic devices for disposable applications, such as simplicity and minimal instrumentation. However, they make the systems either require complicated fabrication process or only provide simple flow pumping. For example, the self-powered capillary microfluidic systems developed by Juncker *et al*. require a complex and expensive fabrication^19–21^. And the surface tension-based passive pumping is dictated by the curvature of the droplets placed at inlets/outlets of microchannels and lacks the abilities to generate constant and well-controlled flow. Other equipment-free pumping techniques, such as evaporation-based pumping^16–18^, gravity-driven flow^22,23^, and finger-actuated pumping^24–26^, are also unable to provide precise and complicated fluidic control.

In our previous work, we developed a “place and play” (PnP) modular polydimethylsiloxane (PDMS) pump^27–29^, which stores pumping energy under vacuum by extracting air from the bulk PDMS. The PnP PDMS pump strategy is very simple: a degassed PDMS pump slab is stored in an air-tight packaging, and the end user only needs to open the packaging and attached the PDMS pump slab on the outlet ports of a microchip before adding liquid samples into the inlet ports. By combining natural capillary action and the PnP PDMS pump, we herein develop a power-free and self-contained microfluidic reactor array (MRA), which autonomously and parallelly performs fluid loading, metering and mixing without external power or complex instruments.

## Results

### System design

**Figure 1:**
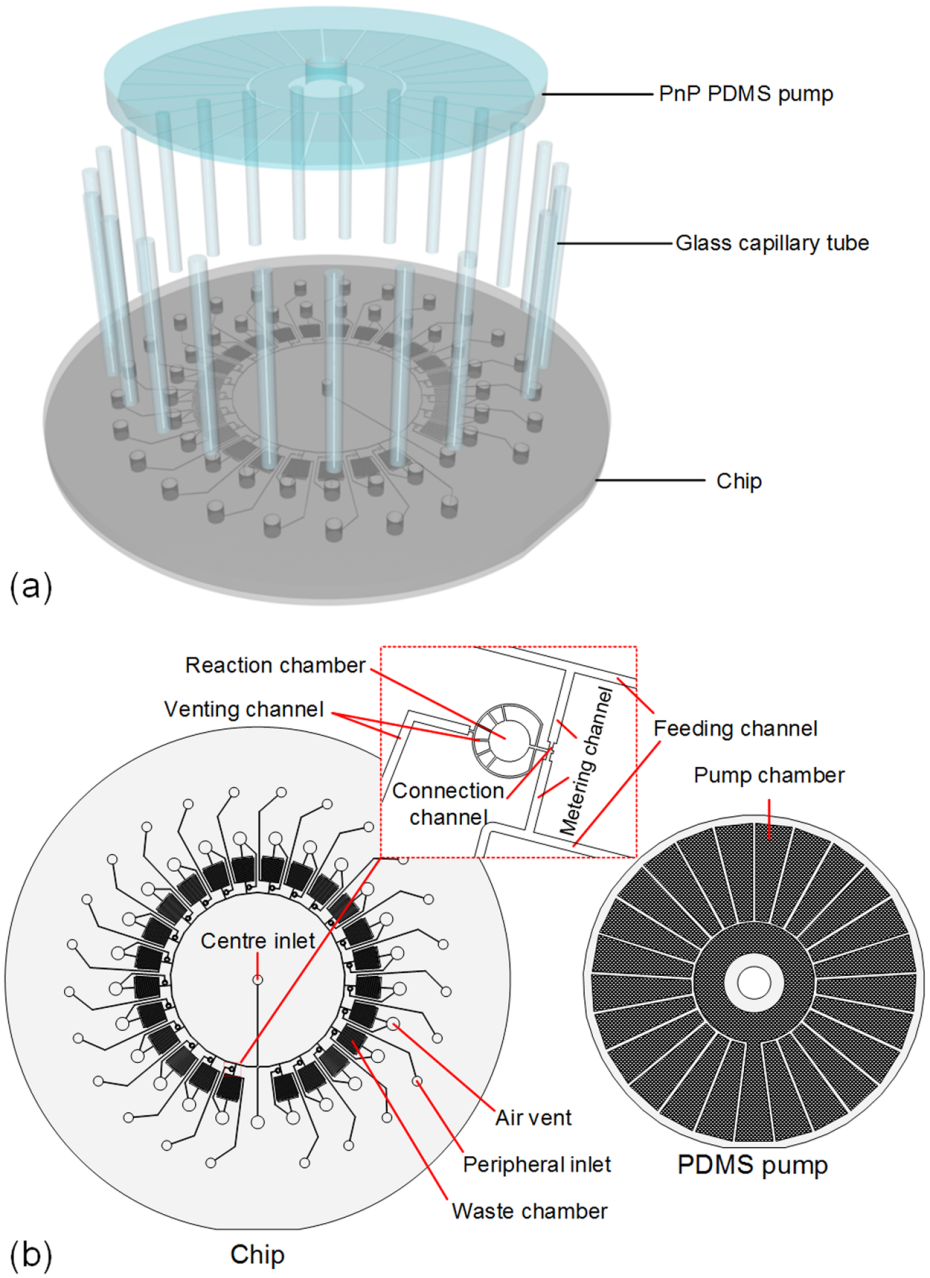
Schematic diagram of the MRA. (**a**) Exploded 3D-view. (**b**) Top view.

A schematic diagram of the proposed MRA is illustrated in Fig. 1. It consists of three major components: an array of glass capillary tubes, a microfluidic chip and a PnP PDMS pump. The capillary tube array is designed to automatically load multiple samples or reagents in parallel *via* capillary action. The microfluidic chip, as shown in Fig. 1b, contains 24 parallel microstructure units that implement 24 simultaneous metering and mixing reactions. Each unit contains a pair of metering structures designed to combine two fluidic samples with a ratio of 1:1. All units are connected with a common feeding channel *via* their respective centre metering channels. In addition, a shallow and narrow connection channel is present at the end of each metering channel. In addition to connecting the metering channel to the mixing channel, the connection channel also acts as a passive stop valve for flow control. The PnP PDMS pump acts as a negative pressure source to drive fluid in the microfluidic chip. Based on our previously reported design^27^, the PnP PDMS pump layout was modified for adapting it to high throughput and highly parallel fluid pumping. Unlike our previous work, where the PnP PDMS pump slab contains 3 independent chambers and can only achieve a pair of liquid plugs metering and mixing, the modular PnP PDMS pump matching with the MRA chip is made of a PDMS slab with 25 independent chambers, which can drive 25 isolated flows and achieve 24 pair of liquid plugs metering and mixing in parallel. Furthermore, each chamber in the PnP PDMS pump contains an array of micro-posts that are designed to increase the air absorption rate (see Supplementary Fig. S1 for more detail). During the operation of the MRA system, each chamber of the PnP PDMS pump corresponds to a venting port of the microfluidic chip and acts as an independent vacuum pump module to suction fluid into a corresponding microfluidic circuit. The working mechanism of the PnP PDMS pump is illustrated in Supplementary Fig. S2. The PnP PDMS pump is degassed *via* vacuum prior to its use. In general, to ensure enough pumping pressure, the PDMS pump slab is activated by maintaining it in a low pressure (~10 kPa) condition for over 2 hr. According to the Hery’s law, dissolved air in the bulk PDMS is removed during vacuum degassing. As a result, vacuum is stored in PDMS substrate of the PnP pump. Once the PnP PDMS pump is removed from vacuum and mounted on a microfluidic chip, the degassed PDMS substrate absorbs air from the environment, which reduces the internal pressure in the channels and chambers (if the open ends of the microfluidic chip are occluded, *e.g*. by liquid samples), and thereby creates a negative pressure to draw liquids into the microfluidic chip.

**Figure 2:**
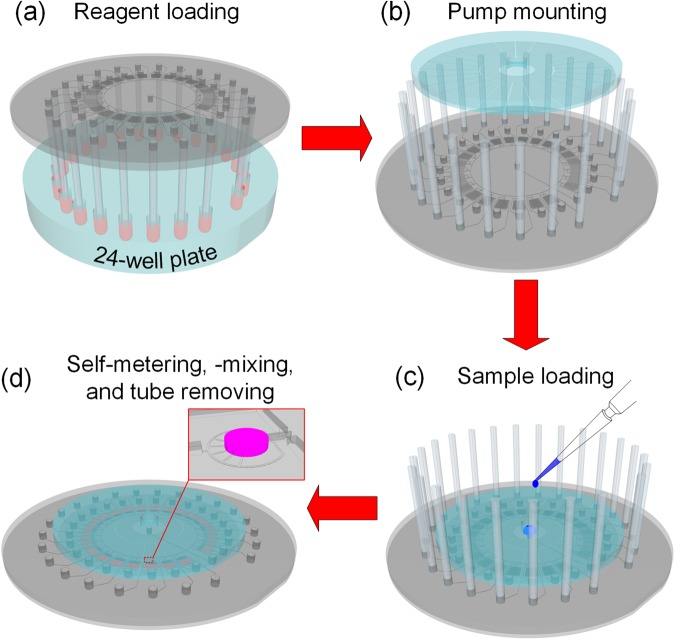
Schematic illustration of the operation of the MRA. (**a**) Parallel loading of different reagents into the microfluidic chip with the capillary tube array, (**b**) mounting of the PnP PDMS pump onto the microfluidic chip, (**c**) formation of a closed microfluidic system after a droplet of sample solution was loaded into the centre inlet port of the microfluidic chip and the aspiration of the liquid into the microchannel under the negative pressure created by the PnP PDMS pump, and (**d**) self metering and mixing of the sample and reagents by the combination of the capillary hydrophobic valves with the dynamic pressure generated by the degassed PDMS pump, and removal of the capillary tubes to facilitate microscopic observation after the completion of self metering and mixing.

A step-by-step procedure for operating the proposed MRA is shown in Fig. 2. First, a PDMS chip mounted with an array of capillary tubes was turned upside down and all tips of the capillary tube array were aligned and immersed in an array of wells filled with reagents (Fig. 2a). Under the action of capillary force, all capillary tubes were spontaneously primed with corresponding reagents. After loading the reagents into the capillary tube array, the MRA chip was turned over and a pre-degassed modular PDMS pump was brought into conformal contact with it, ensuring that each chamber of the pump patch was aligned with each venting port of the MRA chip (Fig. 2b). Then, a drop of liquid sample was pipetted into the central inlet port of the microfluidic chip (Fig. 2c). As soon as all the inlets of the MRA chip were plugged with the liquid, the air supply from the outside of the chip was blocked, and thus a pressure difference was generated between the internal and external parts of the chip because of the absorption of air into the pre-degassed PDMS pump substrate *via* all venting ports of the chip and the surfaces of all PDMS pump chambers^27^. This pressure difference provides the power for driving fluid into the chip. A number of nanoliter-sized droplets can be accurately dispensed and mixed with the aid of specific channels under this degas-induced negative pressure. After the completion of metering and mixing operations, the capillary tubes were removed from the microfluidic chip to allow easy microscope observation (Fig. 2d).

**Figure 3:**
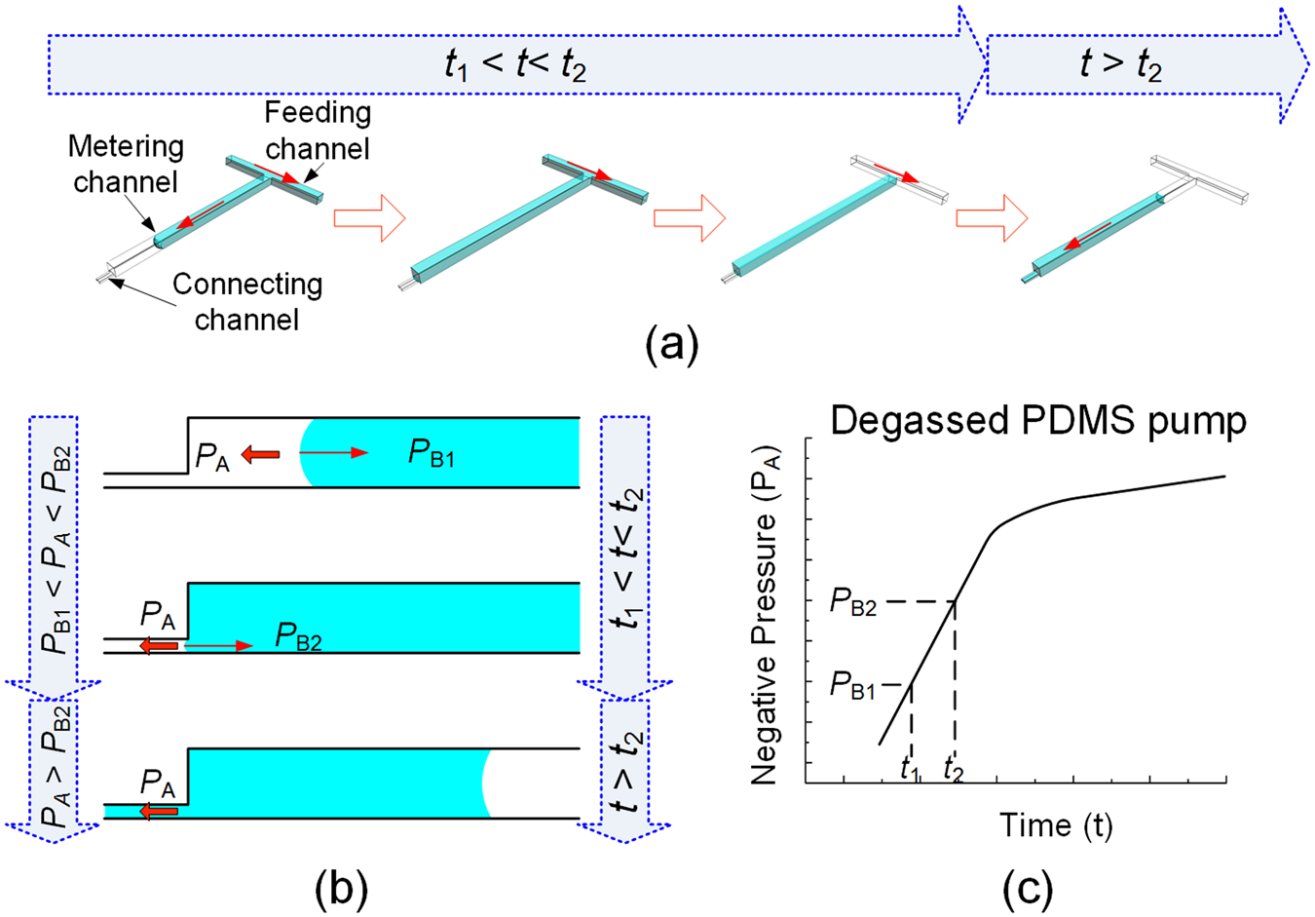
Self metering and mixing principle of the MRA by combining the capillary hydrophobic valving and the degassed PDMS pumping. (**a**,**b**) Time lapse sequences of liquid flow in the microchannel network of the MRA under the action of the capillary hydrophobic valve and degassed PDMS pump. (**c**) Dynamic characteristics of the degassed PDMS pump.

The self metering and mechanism of the MRA is elucidated in Fig. 3, which is dependent on a capillary hydrophobic valve and a growing negative pressure produced by the degassed PDMS pump. Inspired by the work of Yamada *et al*.^30^, a hydrophobic valving was adopted for liquid control in our chip design. That is, there is a pressure barrier (Laplace back-pressure) that must be overcome to drive the fluid flow into a narrow, hydrophobic microchannel. This pressure barrier *P*_B_ can be expressed by the following equation derived from the Young-Laplace equation^31^,

1 $${P}_{{\rm{B}}}=-\,2{\gamma }_{{\rm{la}}}\,\cos \,{\theta }_{{\rm{PDMS}}}(\frac{1}{H}+\frac{1}{W})$$

where *γ*_la_ is the liquid–air interfacial energy (*i.e*., surface tension of liquid) and *θ*_PDMS_ is the equilibrium contact angle between the liquid and material (*θ*_PDMS_ > 90°). *H* and *W* are the depth and width of the rectangular microchannel, respectively. This equation indicates that the pressure barrier will increase as the width or depth of the microchannel decreases. The liquid inside the microchannel moves forward only after the applied pressure *P*_A_ becomes higher than the pressure barrier *P*_B_.

In the proposed MRA, the applied pumping pressure *P*_A_ is created by a degassed PnP PDMS pump. As previously described^27^, the internal pressure in the closed channel system mounted with a degassed PnP PDMS pump gradually decreases over time because the air arrested inside the closed channel system continually diffuses into the degassed PDMS substrate. Accordingly, the pumping pressure, *i.e*., the pressure difference between the atmosphere and internal pressure, is a dynamic pressure and gradually increases with time (as illustrated in Fig. 3c). Next, let us consider the behaviour of liquid in the microchannel network of the MRA. As shown in Fig. 3a, the connection channel is shallower and narrower than both of the feeding channel and the metering channel. From the equation above, it can be seen that the pressure barrier in the connection channel (*P*_B2_) is greater than that in the feeding channel and the metering channel (*P*_B1_). When the liquid is introduced into this structure from the inlet port under the applied pressure *P*_A_ generated by the PnP PDMS pump (*P*_B1_ < *P*_A_ < *P*_B2_), the liquid stops at the entrance of the connection channel because of a larger pressure barrier *P*_B2_ (Fig. 3a,b). After the liquid sample in the inlet port is exhausted, the air is suctioned to remove the excess liquid in the feeding channel, which isolates a precise volume of liquid in the metering channel. As the air arrested in the channels and chambers of the MRA chip is continually absorbed into the degassed PDMS pump substrate, the magnitude of negative pressure in the channels and chambers of the MRA chip increases further and then exceeds the pressure barrier in the connection channel (*i.e*., *P*_A_ > *P*_B2_). As a result, the metered liquid enters into the connection channel and then into the mixing channel to coalesce and mix with another metered liquid (Fig. 3a,b). Obviously, the comparative relationship between the negative pressure (*P*_A_) and the pressure barrier (*P*_B_) is crucial to achieve precise metering and mixing in the MRA. If the geometry and degassing time of PDMS pump are invariant, the comparative relationship between *P*_A_ and *P*_B_ is mainly dependent on the geometry of microchannels, surface tension of liquid and wettability of liquid on PDMS (as indicated in Eqn (1)). Thus, to ensure optimal parallel metering and mixing performance, the key geometrical parameters of the MRA must be properly designed according to the properties of liquids to be metered and mixed (see “S1. Design considerations for the MRA device” in Supplementary Material for more detail).

It must be note that the mixing in each reaction chamber of the MRA relies solely on molecular interdiffusion due to the absence of turbulence. A rough estimate of the characteristic mixing time in reaction chambers of the MRA chip can be obtained by examining the diffusion time across the chamber^32^: *t*_mix_ = *w*^2^/2*D*, where *w* is the radius of the reaction chamber and *D* is the diffusion coefficient of reagent or sample molecules. If considering the radius of the reaction chamber, *w* = 226 μm (in this design) and the diffusion coefficient of protein molecules^33^, *D* ≈ 10^−10^ m^2^/s, the characteristic mixing time is about 4 min.

### Device evaluation

**Figure 4:**
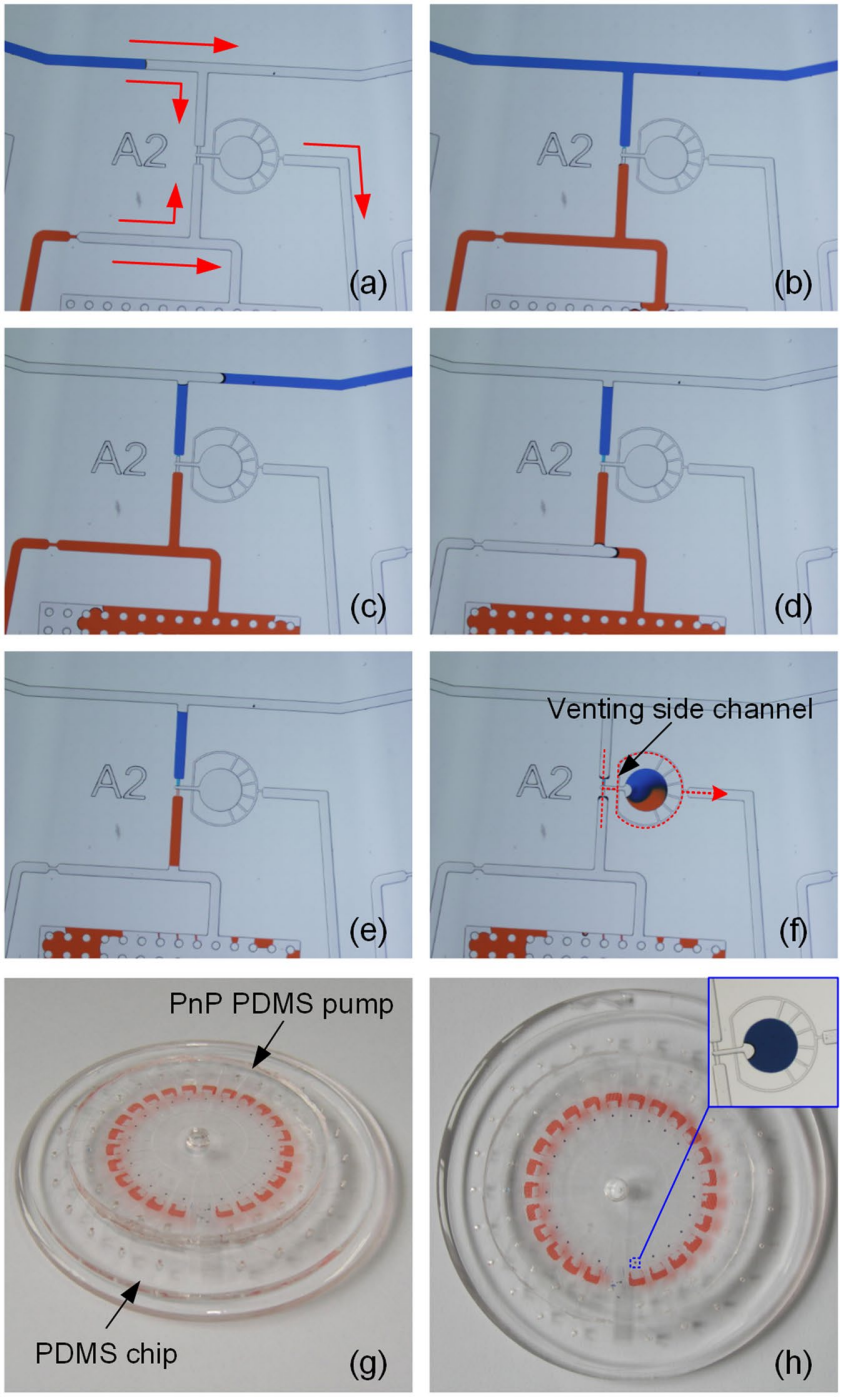
(**a**–**f**) A sequence of close-up photographs of liquid metering and mixing in the MRA. (**a**,**b**) Introduction of dye solution, (**c**), (**d**) and (**e**) metering of dye solution, (**f**) injection and mixing of dye solution in the reaction chamber, the red dotted line indicates the passageway *via* which the PDMS pump chamber opens to atmosphere. (**g**,**h**) Photographs of the MRA after the completion of self metering and mixing.

To demonstrate the functionality of the MRA system, we first tested its self-loading, metering and mixing ability with two dyes. By combining the capillary effect and power-free pumping technique based on the degassed PDMS substrate, a series of liquid-handling operations—loading, metering, and mixing—were carried out in the MRA chip. Figure 4 shows the self metering and mixing process of two dyes in the MRA chip (also see Video S1 in Supplementary Material). When a droplet of sample was placed at the central inlet to block the air flow into the microchannel, the degassed PnP PDMS pump absorbed air in the closed microfluidic circuit, which created a negative pressure within the channels and chambers. As a result, the sample and reagents were aspirated into the microchannels (Fig. 4a). The advancing liquid fronts halted at the entrances of the connection channels because entering the narrower channel must overcome larger Laplace back-pressure. As mentioned above, the negative pressure generated by the degassed PDMS pump is dynamic and gradually increases over time. Thus, there is an interval before the fluid overcomes this pressure barrier and enters into the connection channel. During this interval, the negative pressure created by the PnP PDMS pump continued to pull fluid into waster chambers until the liquids in the inlet ports were exhausted. Then air was pulled into and pushed the excess liquid in the feeding channels to the waster chambers, and thereby droplets with precise volumes were left in the metering channels (Fig. 4c–e). After the completion of metering liquids, the negative pressure produced by the PnP PDMS pump continued to increase. When the pressure exceeded the pressure barrier presented by the connection channel, two metered droplets in each unit entered the connection channel and then coalesced in the mixing channel, and finally rested in the reaction chamber for subsequent detection and analysis (Fig. 4f). To keep the mixed droplet in the reaction chamber for addressable analysis, two venting side channels were designed for each unit to connect the corresponding PDMS pump chamber with the atmosphere and then to allow release of the negative pressure after the completion of selfmixing (as marked by the red dotted line in Fig. 4f). Finally, the capillary tubes were removed from the MRA chip to facilitate microscopic observation, and then a drop of paraffin oil was added into the centre inlet port to seal the mixed droplets and prevent evaporation (see Fig. 4g,h).

When the MRA is applied to the biochemical screening assays, the accuracy and uniformity of metered liquid volume must be ensured because the variation of metered liquid volume can potentially affect the assay results. To investigate the metering consistency and uniformity of the proposed MRA, the microscopic images of the final metered droplets were captured by a CCD camera. The captured images were analysed using an image analysis software (Image-Pro Plus) to determine the droplet area (see Supplementary Fig. S3). The measured area was converted to a corresponding volume by multiplying the value of the reaction chamber depth, 100 μm. Five different MRAs containing 24 units on each chip were tested. The mean volume of the liquid droplets in all reaction chambers was measured to be 16 nL with a CV (coefficient of variation) of 2.5%, which proved that our proposed MRA has reliable accuracy at the nanoliter scale.

**Figure 5:**
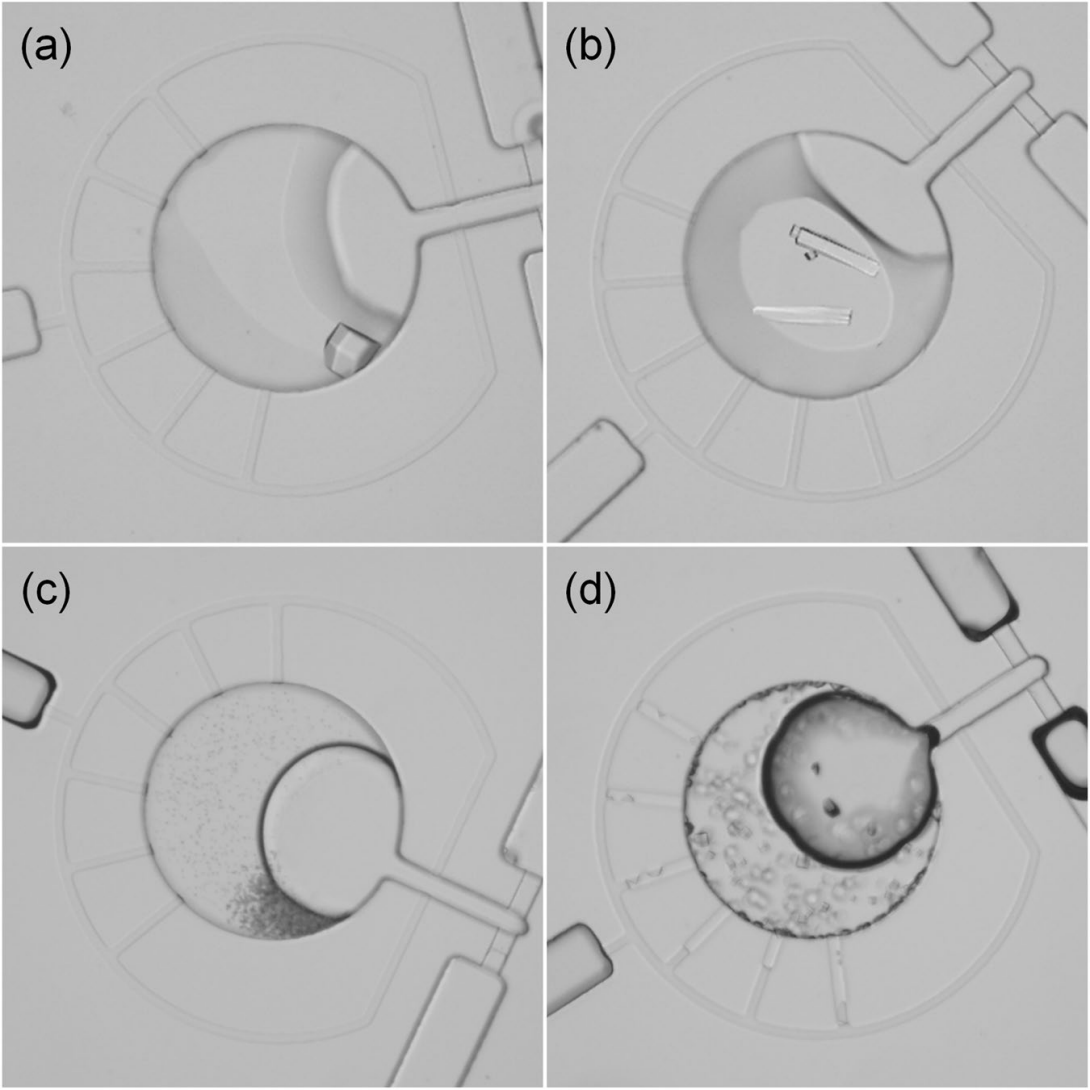
Optical micrographs of typical lysozyme crystallization results obtained with the MRA under different conditions: (**a**) a single tetragonal crystal growing with 0.1 M Sodium acetate trihydrate/2.0 M Sodium formate at pH 4.6 (**b**) the rod-like crystals growing with 0.1 M Sodium acetate trihydrate/8% w/v Polyethylene glycol 4000 at pH 4.6 (**c**) the amorphous precipitate forming with 0.2 M Potassium sulfate/20% w/v Polyethylene glycol 3350 (**d**) a large number of smaller tetragonal crystals growing with 0.2 M Magnesium nitrate hexahydrate/20% w/v Polyethylene glycol 3350.

To evaluate the feasibility of the MRA for biochemical screening, we further applied it to the screening of protein crystallization conditions. The MRA proposed in this study can easily be used to establish a microbatch protocol for protein crystallization. With the parallel, discrete nanoliter microfluidic system, the traditional microbatch protocol is simplified—multiple precipitants are loaded by the capillary force simultaneously and automatically; the protein sample is introduced by a single pipetting, and the protein sample and multiple precipitants are metered, dispensed, and mixed using a combination of capillary hydrophobic valving and degassed PDMS pumping, producing an array of droplets with each droplet representing an independent crystallization experiment. Lysozyme, a representative model protein, was chosen as a test sample. Based on the liquid operation procedure mentioned above, lysozyme solution and precipitants were dispensed and mixed in reaction chambers for incubation. Crystal growth was observed in the MRA (Fig. 5) and showed an excellent degree of correlation with successful conditions revealed by conventional screening techniques. Here, crystals were obtained in 16-nL droplets using the microbatch method. The size of crystals obtained with appropriate crystallization conditions is typically ~100 µm, which is large enough for X-ray diffraction studies.

## Discussion

Taking advantage of capillary action as a parallel loading pressure source, a pre-degassed PDMS slab as a fluid-driven pressure source and microchannel contractions as passive valves, we have developed a self-contained and power-free microfluidic platform for biochemical screening. This platform is an easy-to-use and inexpensive solution for automated liquid sample manipulation on the nanoliter scale. Based on the combination of capillarity action and degassed PDMS pumping, this device can automatically manipulate the motion of liquids, performing priming, metering, dispensing, and mixing. In this device, a single injection of liquid is sufficient to prepare multiple nanoliter-sized aliquots for different reactions, and then performs multiple analyses or screening without any external pumping or valving mechanisms. We have also demonstrated the feasibility of this microdevice in biochemistry applications by using it to screen protein crystallization conditions. Due to its low cost and sample consumption, ease of operation, and high portability, this simple PnP platform can potentially be used for multiple (bio)chemical analyses and high throughput screening in individual laboratories or remote locations.

## Methods

### Device fabrication

Devices were fabricated using soft lithography^34^. Briefly, SU-8 (MicroChem, Newton, MA, USA) masters were fabricated by standard photolithography on 3′′ silicon wafers. We created separate masters for the PDMS pump and chip sections of the device. The microfluidic chip layer was prepared using a multi-layer SU-8 process, and consisted of three levels of features: 100-µm-high features for the feeding channels, the metering channels and the reaction chambers; 15-µm-high features for the connection channels; 5-µm-high features for the venting channels. The PDMS pump master is a single-layer mold with 100-µm-high features. After the completion of the master molds, patterned PDMS layers were fabricated using a procedure developed previously^35^. Briefly, PDMS (Sylgard 184, Dow Corning) was mixed with a ratio of 10:1 of base to cross-linking agent, and was casted onto the master with a holding frame. A PET film was placed on the top of the frame filled with PDMS prepolymer to fabricate a double-sided flat PDMS pad. After curing at 90 °C for 1 h, the two molded PDMS slabs were peeled away from the masters. Then, the PDMS chip layer was punched with a sharpened needle to create inlet/outlet ports and bonded to a 3′′glass wafer using oxygen plasma treatment. After bonding, 24 capillary tubes (inner diameter: 0.2 mm, outer diameter: 2 mm, length: 20 mm) were inserted into 24 inlets of the chip, respectively (see Supplementary Fig. S4). The PDMS pump layer was trimmed with a razor. Before use, the PDMS pump layer was placed under vacuum for 1 h at 10 kPa and then sealed in airtight packaging for ready-to-use^27^.

### Crystallization experiments

For the protein crystallization experiments described here, lysozyme was used as model protein to investigate the feasibility of the MRA chip in protein crystallization. Hen egg white lysozyme protein (J&K Scientific Ltd, Beijing, China) was dissolved in 0.02 M sodium acetate buffer at pH 4.6 to obtain a protein stock solution of concentration 40 mg/mL. A set of 24 precipitants was used to screen the crystallization conditions. All precipitants were prepared from stock solutions purchased from XtalQuest Company, Limited (Beijing, China). The entire list of 24 precipitants can be found in Supplementary Table S1. Firstly, a parallel loading of 24 precipitants was carried out by immersing the tips of the capillary tube array mounted on the MRA chip into an array of 24 wells filled with crystallization precipitant solutions. Then, the MRA chip was turned over and a pre-degassed PnP PDMS pump was aligned and attached to its top surface. After mounting the PnP PDMS pump, a droplet of protein solution was added into the center inlet of the MRA chip by a micropipette. Once the introducing and mixing of the protein and the crystallization reagents was completed, a few drops of paraffin oil were added into the center inlet to seal the mixture droplets, suppressing the evaporation during incubation. Finally, the MRA chip was incubated in a 4 °C refrigerator and monitored periodically using an inverted microscope (IX-51, Olympus, Tokyo, Japan) equiped with a CCD camera (DP70, Olympus, Tokyo, Japan).

## Electronic supplementary material

Supplementary Information

Video
