# Supplementary material for: A power-free, parallel loading microfluidic reactor array for biochemical screening
Source: Sci Rep. 2018 Sep 12;8:13664. doi: 10.1038/s41598-018-31720-y (PMC6135844; doi:10.1038/s41598-018-31720-y)
Supplement: Supplementary file 1 — Supplementary Information [file 41598_2018_31720_MOESM1_ESM.pdf]

Supporting Information

Title: A Power-Free, Parallel Loading Microfluidic Reactor Array for Biochemical Screening

Yanwu Liu[1] & Gang Li[2*]

[1] College of Stomatology, Chongqing Medical University, Chongqing 401147, China.

[2] Defense Key Disciplines Lab of Novel Micro-Nano Devices and System Technology, Key Laboratory of Optoelectronic Technology and Systems, Ministry of Education, Chongqing University, Chongqing 400044, China

*Correspondence and requests for materials should be addressed to G. Li. (email: gang_li@cqu.edu.cn)

S1. Design considerations for the MRA device

As illustrated in Figure 3 of this paper, the self-metering and self-mixing mechanism of the MRA is dependent on a capillary hydrophobic valve and a growing pumping pressure produced by the degassed PDMS pump. Here, we combine the geometry and wettability of the channel in the chip with the effective surface area and degassing time of the PDMS pump in such a way that the liquid is transported according to a "programmed" succession of "flow" and "stop" actions. By properly controlling the time periods of "flow" and "stop" actions, effective and accurate metering and mixing for liquids can be achieved. In the MRA device, the connection channel is designed to be smaller than the feeding/metering channel and has higher Laplace back-pressure for water, so the joint of the connection channel and the metering channel plays the role of a valve. In addition, owing to the dynamic process of the diffusion of air into PDMS pump slab from the closed channel, the magnitude of negative pressure in the channel gradually increases with time [S1]. The dynamic variation of negative pressure in the MRA includes two phases: (i) all the chambers of PDMS pump together absorb the air trapped in the closed channel network to decrease the internal pressure and thereby draw the liquids loaded on the inlet ports into the feeding/metering channels; (ii) after the common feeding channel is filled with the liquid, each pump chamber continues to absorb the air arrested in the corresponding channel/chamber unit, which results in a further increase in the magnitude of negative pressure in each corresponding microfluidic circuit until the pumping pressure in each channel/chamber unit surpasses the burst pressure of the corresponding stop valve. Thus, there is a delay interval between the time the liquid fills the feeding/metering channel and the time at which the liquid bursts into the mixing channel. If the excess liquids in all the feeding channels are removed within this interval, the precise parallel metering and mixing can be achieved for the MRA. In other words, in order to achieve precise metering and mixing, the duration of filling liquids in the feeding/metering

channels and removing the excess liquids in the feed channels must be shorter than the growth period of the pumping pressure in the device from P_{B1} to P_{B2}. If the geometry and degassing time of the PnP PDMS pump are invariant, that is, the dynamic growth behavior of P_A is kept unchanged, the performance of MRA is mainly dependent on valve geometry, device material and fluid properties. For the MRA described in this paper, the pressure barriers in the feeding channel and the connection channel can be roughly calculated according to equation (1) in the paper. For deionized water (γ = 70 mN/m) and PDMS-based device (θ_{PDMS} = 105°), a feeding channel with cross section of 100 μm × 100 μm (width × depth) has a barrier pressure (P_{B1}) of 725 Pa, and a connection channel with cross section of 40 μm × 15 μm (width × depth) has a barrier pressure (P_{B2}) of 3324 Pa. For the PnP PDMS pump described in this paper, the total internal air absorption area is about 2700 mm^2, and each individual pump chamber has an air absorption area of about 98 mm^2, except for the center pump chamber with an air absorption area of about 350 mm^2. According to our previous work[S1], the time period needed for increasing the magnitude of the negative pressure in each channel/chamber unit of MRA from 725 Pa to 3324 Pa is 3–4 min. For the MRA described in the paper, in general, 1 μL sample and 0.5 μL reagent are loaded on the center inlet port and each peripheral inlet port, respectively. In such a case, about 0.8 μL excess liquid is required to be drained along a flow path length of 99 mm from the center inlet port to the main outlet port and 0.3 μL excess liquid is required to be drained along a flow path length of 17 mm from each peripheral inlet port to the corresponding outlet port. Based on a rough calculation[S2], it takes about 90 s to drain 0.8 μL liquid from the common feeding channel and about 80 s to drain 0.3 μL liquid from each peripheral feeding channel. Thus, the removing time of the excess liquids in the feeding channels is shorter than the growth period of pumping pressure in the device from P_{B1} to P_{B2}, thereby allowing an enough time margin for liquids metering.

As mentioned above, the main contribution to time margin for precise metering originates in the stop valve for this MRA. Therefore, if the MRA is desired to parallelly meter and mix liquids whose surface tension and contact angle on PDMS are significantly different from those of water, the cross-section of the connection channel can be tailored to ensure enough time margin for effectively metering liquids.

Reference

S1. Li, G., Luo, Y., Chen, Q., Liao, L. & Zhao, J. A "place n play" modular pump for portable microfluidic applications. *Biomicrofluidics* **6**, 014118 (2012).

S2. Bruus, H. *Theoretical microfluidics*. 47-50 (Oxford university press, 2008).

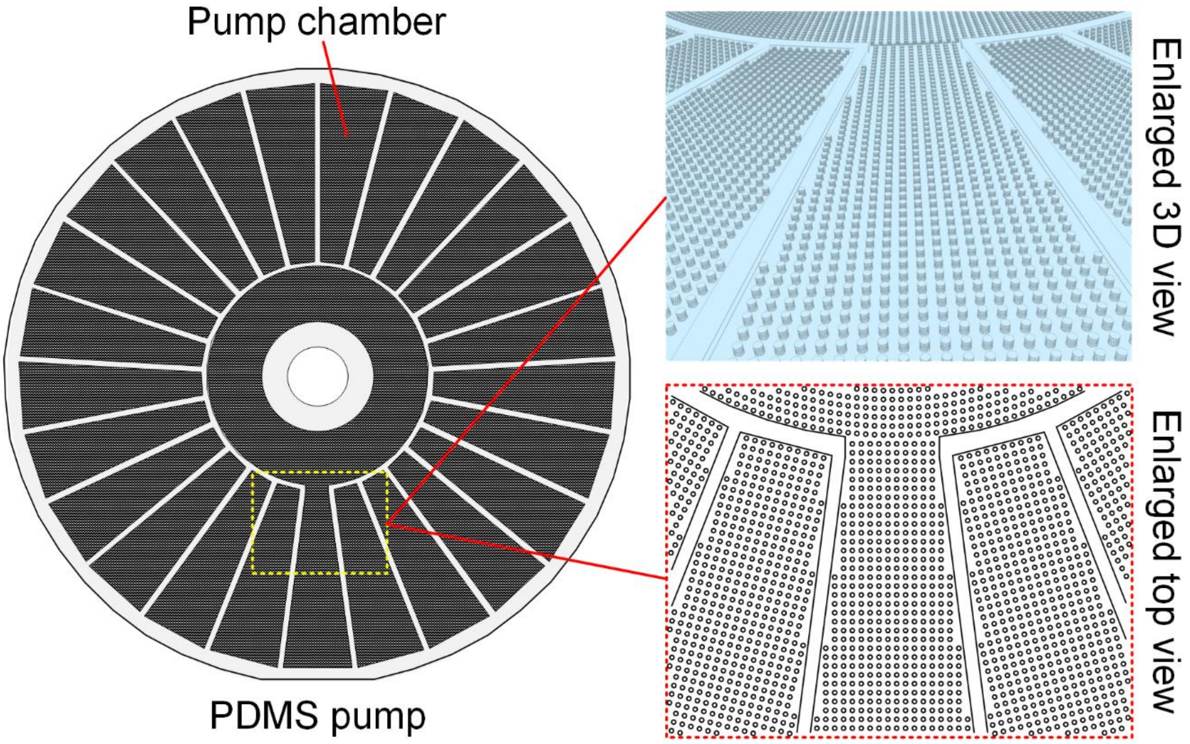

Figure S1. Schematic diagram of the PnP PDMS pump.

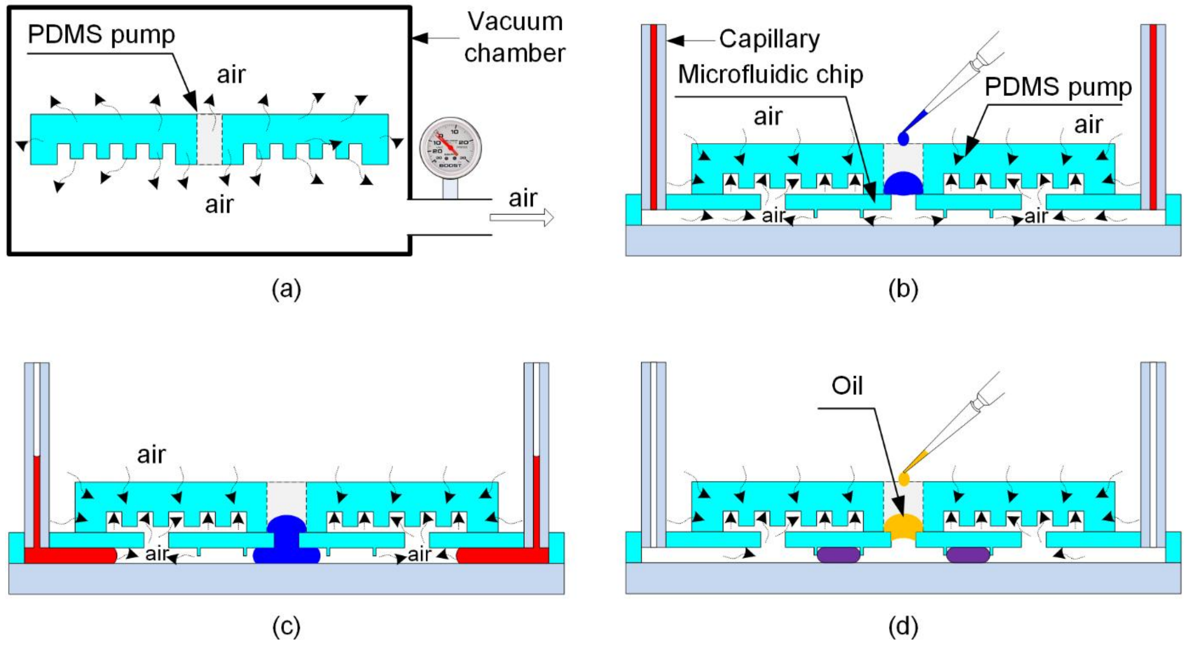

Figure S2. Schematic illustration of the working mechanism and operation of the PnP PDMS pump: (a) the degassing of the PnP PDMS pump in a vacuum chamber, (b) the mounting of the PDMS pump on a MRA chip and the redissolving of air into the PDMS pump from atmosphere, (c) the formation of the closed microfluidic system after a droplet of liquid was loaded into the inlet, and the aspiration of the liquid into the microchannel under the negative pressure created by the modular PDMS pump, (d) the oil-sealing of the mixture droplets for minimizing the evaporation.

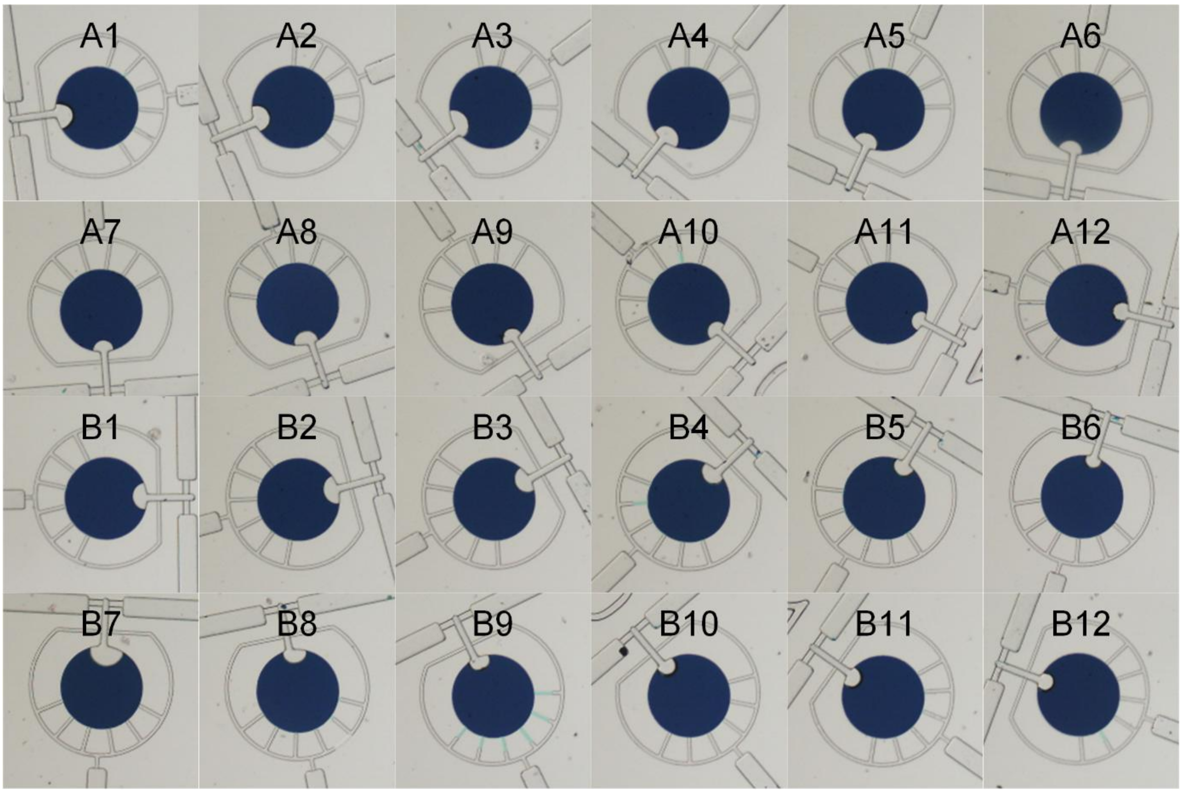

Figure S3. Optical micrographs of the metered droplets in all 24 reaction chambers of a representative MRA chip.

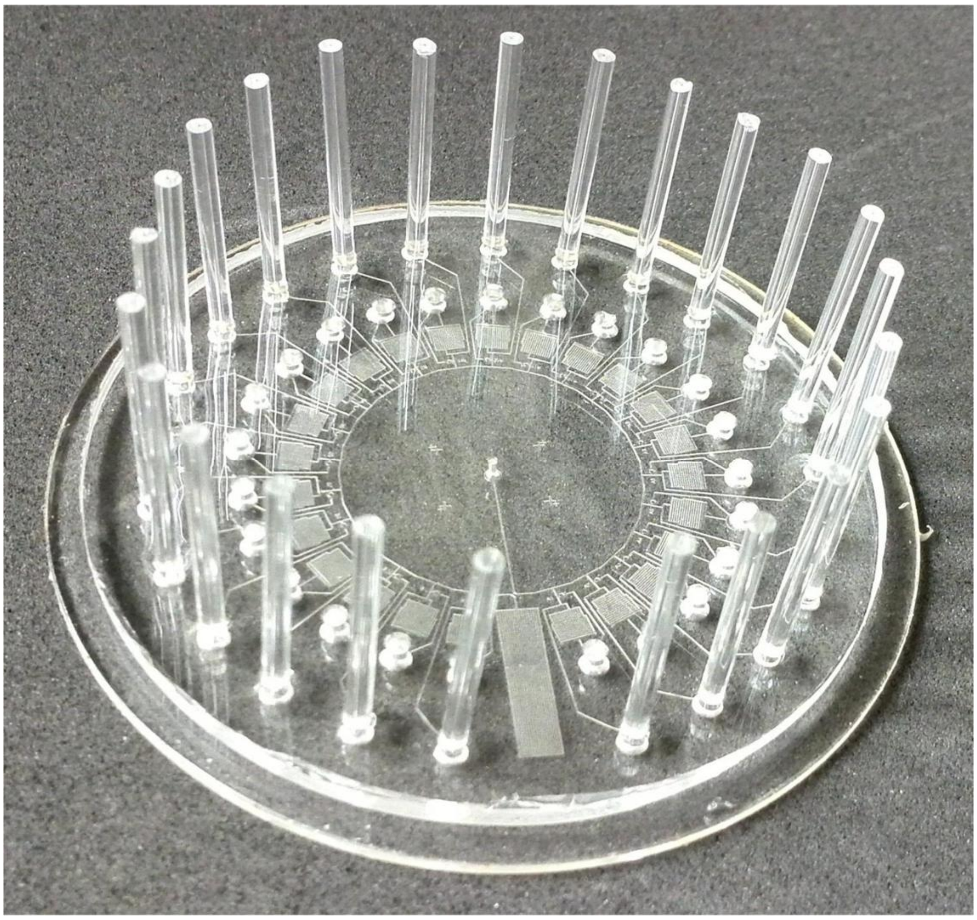

Figure S4. Photograph of a MRA chip mounted with capillary tubes.

Table S1. The crystallization conditions and the screening results of lysozyme using the MRA chip presented in the main text.

Condition index	Screening condition	Screening Result
1	0.02 M Calcium chloride dihydrate, 0.1 M Sodium acetate trihydrate pH 4.6, 30% v/v (+/-)-2-Methyl-2,4-pentanediol	Crystals (tetragonal)
2	0.4 M Ammonium phosphate monobasic	Clear drop
3	0.1 M TRIS hydrochloride pH 8.5, 2.0 M Ammonium sulfate	Clear drop
4	0.2 M Sodium citrate tribasic dihydrate, 0.1 M HEPES sodium pH 7.5, 30% v/v (+/-)-2-Methyl-2,4-pentanediol	Clear drop
5	0.2 M Magnesium chloride hexahydrate, 0.1 M TRIS hydrochloride pH 8.5, 30% w/v Polyethylene glycol 4,000	Crystals (tetragonal)
6	0.2 M Ammonium acetate, 0.1 M Sodium acetate trihydrate pH 4.6, 30% w/v Polyethylene glycol 4,000	Crystals (needle-like)
7	0.2 M Magnesium chloride hexahydrate, 0.1 M HEPES sodium pH 7.5, 30% v/v Polyethylene glycol 400	Crystals (rod-like)
8	0.2 M Calcium chloride dihydrate, 0.1 M Sodium acetate trihydrate pH 4.6, 20% v/v 2-Propanol	Crystals (tetragonal)
9	0.1 M Imidazole pH 6.5, 1.0 M Sodium acetate trihydrate	Crystals (tetragonal)
10	0.2 M Ammonium sulfate, 30% w/v Polyethylene glycol 8,000	Crystals (rod-like)
11	2.0 M Ammonium sulfate	Clear drop
12	4.0 M Sodium formate	Clear drop
13	0.1 M Sodium acetate trihydrate pH 4.6, 2.0 M Sodium formate	Crystals (tetragonal)
14	0.1 M TRIS hydrochloride pH 8.5, 8% w/v Polyethylene glycol 8,000	Crystals (tetragonal)
15	0.1 M Sodium acetate trihydrate pH 4.6, 8% w/v Polyethylene glycol 4,000	Crystals (rod-like)
16	0.1 M HEPES sodium pH 7.5, 10% v/v 2-Propanol, 20% w/v Polyethylene glycol 4,000	Crystals (tetragonal)

17	0.05 M Potassium phosphate monobasic, 20% w/v Polyethylene glycol 8,000	Clear drop
18	0.2 M Magnesium formate dihydrate	Clear drop
19	0.1 M Sodium acetate trihydrate pH 4.6, 2.0 M Sodium chloride	Crystals (tetragonal)
20	0.1 M MES monohydrate pH 6.5, 12% w/v Polyethylene glycol 20,000	Crystals (tetragonal)
21	0.1 M HEPES pH 7.5, 20% w/v Polyethylene glycol 10,000	Microcrystals
22	0.2 M Lithium chloride, 20% w/v Polyethylene glycol 3,350	Crystals (tetragonal)
23	0.2 M Magnesium nitrate hexahydrate, 20% w/v Polyethylene glycol 3,350	Microcrystals
24	0.2 M Potassium sulfate, 20% w/v Polyethylene glycol 3,350	Precipitate

Legends of Video

Video S1: Demonstration of the self-metering and self-mixing process of two dyes in the MRA chip.
